# Design Guidelines for a Technology-Enabled Nutrition Education Program to Support Overweight and Obese Adolescents: Qualitative User-Centered Design Study

**DOI:** 10.2196/14430

**Published:** 2019-07-29

**Authors:** Cynthia LeRouge, Polina Durneva, Savitha Sangameswaran, Anne-Marie Gloster

**Affiliations:** 1 Health Informatics and Analytics Program Department of Information Systems & Business Analytics Florida International University Miami, FL United States; 2 Department of Biomedical Informatics and Medical Education School of Medicine University of Washington Seattle, WA United States; 3 Nutritional Sciences Program School of Public Health University of Washington Seattle, WA United States

**Keywords:** healthy eating, user-computer interface, consumer health informatics, adolescent, obesity, overweight, nutrition, cooking, user-centered design

## Abstract

**Background:**

Childhood overweight and obesity are major health challenges in the United States. One of the recommendations to combat obesity is to maintain a healthy diet, which is often best supported by eating home-cooked meals to control cooking methods, ingredients, and portions. Diet control through home cooking is challenged because of the decline in culinary skills in the population and a paucity of effective culinary nutrition education (CNE) programs. Providing technology-enabled CNE (CNE-tech) to overweight and obese adolescents can equip them with life skills that can assist them in the future. Such skills can facilitate saving money, eating healthier, and creating social environments. In addition, CNE builds cooking confidence and food literacy that in turn can build adolescent self-efficacy, particularly toward managing their health behaviors.

**Objective:**

This study aimed to inform functionalities, design requirements, and the context of use for CNE-tech that could enhance overweight and obese adolescents’ healthy food literacy, cooking confidence, and general self-efficacy with regard to self-management to ultimately promote healthy lifestyle management.

**Methods:**

The design science study was completed in 2 distinct phases engaging overweight and obese adolescents, parents of overweight and obese adolescents, and the health care providers that treat adolescents with these conditions. Phase 2, our primary source of data, involved user-centered design methods including the following: (1) early stage prototype usability analysis, (2) semistructured interviews with 70 overweight or obese adolescents engaged in a healthy behavior program, and (3) semistructured interviews with 10 health care providers. Data were analyzed using constant comparison analysis to identify functionalities, design requirements, and inform the context of use of CNE-tech.

**Results:**

Data revealed specific desired functionalities for the CNE-tech related to building cooking skills, populating a healthy recipe database, suggesting healthy alternatives, supporting the construction of a healthy plate, and the ability to share healthy recipes and cooking accomplishments. Moreover, the adolescents provided design requirements pertaining to the presentation (eg, vivid colors, semirealistic images, and cooking sounds), use of multimedia, and gaming. Data further revealed contextual factors, such as shared experiences with family members and enhanced continued use.

**Conclusions:**

We demonstrate the potentiality of creating CNE-tech that could effectively lead to better self-care and induce sustainable behavioral change as it facilitates skill building, self-efficacy, and a pathway that enables overweight and obese adolescents to influence cooking habits in their family home and future dwellings. Our CNE-tech–proposed solution aligns with the goals of overweight and obese adolescents and also reflects existing theories about behavioral change.

## Introduction

### Background

Overweight and obesity are major public health issues affecting 12.7 million children and adolescents [1-3] and the overall health care system, resulting in the annual cost of about US $190 billion in the United States [4]. To date, it has proved to be challenging to isolate and study the effects of single factors (genetic and nongenetic) on this condition, as obesity and overweight seem to be caused by the interplay of a variety of factors [5]. Lack of physical activity, unhealthy eating, or the combination of the two is a primary cause of overweight and obesity in children and adolescents [6]. Genetic predisposition and certain social factors (eg, low socioeconomic factors and physical environment) are also associated with different levels of obesity and overweight [7].

Healthy diet and physical activity are crucial to combat these conditions and maintain healthy weight. Studies have shown that enhanced culinary skills could lead to better diet quality over a number of years as more home-cooked meals would be consumed [8]. Consumers in the United States, unfortunately, have been experiencing a decline in culinary skills [9] and been shifting from home-cooked meals to prepared foods. Previous studies explored such shift with respect to wide availability and low prices of convenience foods [10] and consumers’ socioeconomic status, culinary experience, free time, and limited culinary abilities [11-15].

Healthy culinary habits and behaviors have been previously explored, defined, and associated with positive health outcomes [16]. Educational programs and health interventions can, therefore, be effective and important for promoting healthy eating. Traditional nutrition education programs have, however, been shown to be ineffective in the past because of the content taught in such classes [17,18]. These programs focus primarily on educating people about food characteristics and potential medical disorders associated with over or underconsumption of nutrients [19,20] and do not provide typical consumers with skills necessary to prepare and select healthy meal options. Culinary nutrition education (CNE), on the contrary, can be more advantageous by bringing knowledge of healthy eating into action.

CNE programs teach students how to adequately nourish themselves through food, convert raw ingredients into edible healthy dishes, and maintain healthy diet habits in the future [21]. Through CNE, adolescents benefit by increasing food literacy and cooking confidence [22], which have been described as inadequate among the youth [23]. Food literacy can be defined as “the ability to make healthy food choices by having the skills and knowledge necessary to buy, grow, and cook food with implications for improving health” [24]. Cooking confidence refers to confidence in cooking certain meals, implementing various cooking techniques, and following recipes [25]. In addition to potential health benefits, increased cooking confidence and food literacy can build adolescents’ self-efficacy [22] to maintain healthy eating behaviors. Knowledge gained through cooking can also help adolescents make informed less calorie-dense choices when choosing to prepare a recipe or when eating at a restaurant [26]. In addition, by influencing meal choices and taking over cooking responsibilities, adolescents may serve as catalysts for healthy eating in their families. Adolescents with high food literacy can build stronger connections with their families, according to Utter et al [27].

Despite their obvious benefits, nutritional health intervention programs are not widely available for US adolescents [28]. One of the ways to address such problem is to integrate lifestyle intervention through information and communication technologies (ICTs) that can provide tailored education in a cost-effective manner. A recent systematic review on the use of nutritional interventions for adolescents using ICTs indicated that game-based ICTs, which focused on promoting healthy habits of adolescents, tended to be quite efficient in their purposes and that “long-term interventions for adolescents that make use of frequent exposure to technological resources, and that have a theoretical component aimed at a single health behavior change, tend to be more successful” [29]. Regarding specific ICTs, improvements in lifestyle habits, such as smoking cessation, have been shown to be positively associated with an increase in mobile text messaging, a type of ICT intervention [30]. An ICT-based intervention in the form of mobile apps seems to be quite popular. There currently exist 70,000 apps targeting people with various medical conditions (eg, an app to manage asthma symptoms) and health goals (eg, fitness and nutrition apps) [31]. Even though the importance of current diet mobile apps (eg, apps focusing on calorie intake) is hard to underestimate, it is plausible that apps related to CNE tailored to an adolescent population might play a role in the process of diet improvement.

Among all age groups, adolescents are typical early adopters of technology because of peer pressure, self-efficacy, and self-innovativeness [32]. Findings based on a nationally representative sample of adolescents indicate that the majority of adolescents use the internet to find health-related information [33]. Moreover, it has been shown that about 95% of adolescents in the United States, irrespective of gender, race, ethnicity, and socioeconomic background, either own or have access to a mobile phone [34]. Given potentially high interest, reach, a favorable adoption environment, convenience benefits related to time and location (eg, fitting cooking in the adolescents’ busy schedules), and an increased call from the field of diabetes to include more tech solutions for providing nutrition care [35-37], a technology-enabled solution to assist adolescents with cooking would be highly desirable. There is, however, a dearth of evidence-based research that focuses on CNE-related mobile apps, particularly for adolescents [35].

A literature review of mobile apps for weight management by Azar et al found that out of 200 top ranked health and fitness apps (23 eligible apps) only 3 were focused on healthy cooking [38]. Furthermore, in our own search for commercially available apps for cooking on the iTunes App Store, we found about 100 relevant results almost all of which were related to cooking games. Most cooking and food related games tend to be centered around the entertainment value (eg, cooking or preparing food in virtual worlds) [39]. Even though the efficacy of such apps to teach cooking skills is yet to be evaluated, some preliminary studies did indicate that games may increase children’s intake of fruits and vegetables [40]. Although the preliminary studies may be promising, unfortunately, it also seems that most of the commercially available apps do not address all components of CNE, particularly the knowledge about intake of fruits and vegetables, sodium, and added sugar, and are not tailored to the adolescent audience [41].

### Objective

Recent mobile health studies indicate a tendency to implement user-centered design (UCD) iterative technology development because of its deep focus on determining users’ needs and the environment in which a technology might be used [42]. Mobile health interventions that are centered around a user or patient are highly responsive to users’ preferences and, as a result, can induce higher level of engagement and smoother adoption [43,44]. Given the potential and nuances of the adolescent population, we root our effort in a UCD (and in some ways user-driven) approach to explore the design, functionalities, and potential of a technology-enabled solution to facilitate overweight and obese adolescents’ self-care and provide them with CNE. The objectives of the study are to identify (1) user-centered functionalities (ie, a set of capabilities associated with a software [45]), (2) design requirements for a CNE technology (CNE-tech), and (3) contextual factors that could affect the adoption and use of technology.

Our study contributes to the existing literature by addressing the dearth of evidence-based research on CNE that leverages technology. We will also discuss our results in light of existing behavior change theories.

## Methods

### Study Design

In this design science study, we applied principles of UCD. We selected a UCD approach because it focuses on usability of the final technology product as demonstrated through learnability, efficiency, memorability, and satisfaction [46]. We identified multiple facets of usability of a mobile app–based intervention for overweight and obesity that aligned with the needs of our end users, adolescents. The study was guided (design and protocols) by constructs from the Unified Theory of Acceptance and Use of Technology (UTAUT)–performance expectancy (likelihood of meeting goals with Consumer Health Technology), effort expectancy (ease of use), and social influence (influence by others in the use of technology) [47].

**Figure 1:**
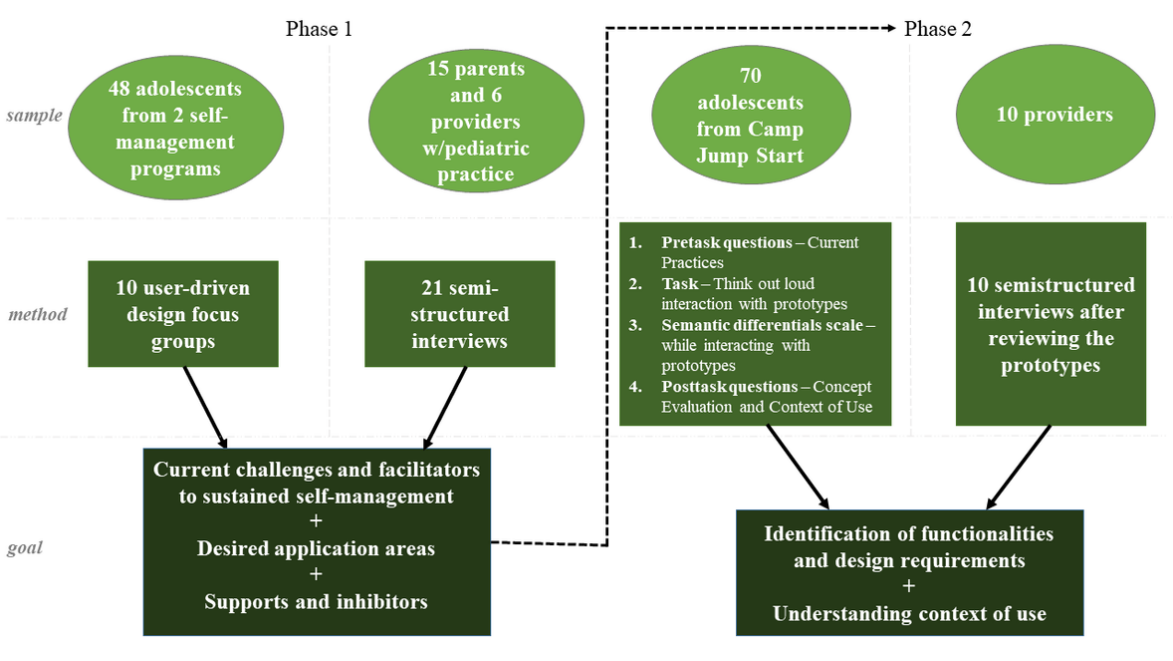
Multiphase research design.

Our user-centered study comprised 2 mixed-methods phases with phase 2 serving as the primary source of data for this paper. Figure 1 provides details on sample, method, and goal of each phase. The study protocol for both phases was approved by the Institutional Review Board of Saint Louis University. The adolescents were asked to assent before all data collection sessions. Reporting of qualitative data was guided by the Consolidated Criteria for Reporting Qualitative Research [48].

### Phase 1

The goal of this phase was to detect desired app areas to assist with self-management as well as to identify design requirements and functionalities for such areas. It was also important to determine the context of self-management for the adolescents.

Focus groups in phase 1 focused on identifying possibilities for sustaining self-management, overcoming challenges and enhancing facilitators, conceptualizing desired app areas, and contextual supports and inhibitors to using technology. More detailed information about participants of the focus groups, criteria of selection, description of the protocol, and adolescents’ initial feedback can be found in the study by Knoblock-Hahn et al [49]. Following focus groups with the adolescents, semistructured interviews with the parents and health care providers with pediatric practice took place and addressed the following topics: awareness of the adolescent’s weight, barriers and facilitators for treatment of adolescent overweight and obesity, and perceived usefulness of and intent to use technology. More detailed information about semistructured interviews can be found in the study by Knoblock-Hahn et al [50]. As a result of the focus groups and semistructured interviews, 5 viable app areas were identified: (1) social networking, (2) motivation, (3) cooking (which the adolescents also referenced as *recipe builder*), (4) physical activity management, and (5) food management. Midfidelity wireframe screen design prototypes [51] were developed based on phase 1 data collection.

### Phase 2

Phase 2 was conducted to evaluate the adolescents’ response to user-inspired prototypes of the app areas identified in phase 1 and detect more specific functionalities and design requirements pertaining to each of the 5 app areas. We recruited 70 adolescents from Camp Jump Start [52], a recognized, evidence-based adolescent weight loss summer camp program (led by a medical provider) [53]. Participants of the camp were aged between 9 and 18 years coming from 50 states and 23 countries. Some of the participants were from homeless and impoverished families, whereas others were coming from families with high socioeconomic status. Most of the participants were from the middle-class families.

Our study included a diverse group of black, white, and Hispanic adolescents from various socioeconomic backgrounds. Both males and females were included in the study. The age of the participants ranged from 12 to 17 years. The inclusion criteria were based on the age, computer use, and body mass index (participants were in 85^th^-99^th^ percentile range). The adolescents completed a series of activities covering from 2 to 4 of the 5 app areas identified in phase 1, as time allowed. We used a randomized *round-robin* rotation of the order of app areas introduced to each participant to assign app areas to 70 participants. This rotation was used to reduce any bias that may have resulted in the sequencing of viewing app areas and to facilitate coverage of all the apps with the aggregated pool of participants within the time constraints of each participant usability session. As a result of this rotation, 15 participants reviewed the CNE prototypes (n=15). For purposes of this paper, we also reviewed and included relevant data from the participants who reviewed other app areas for comments and references related to their current use of technology, cooking references, and general contexts of using technology to support their self-management.

#### Prototype Usability Testing

**Figure 2:**
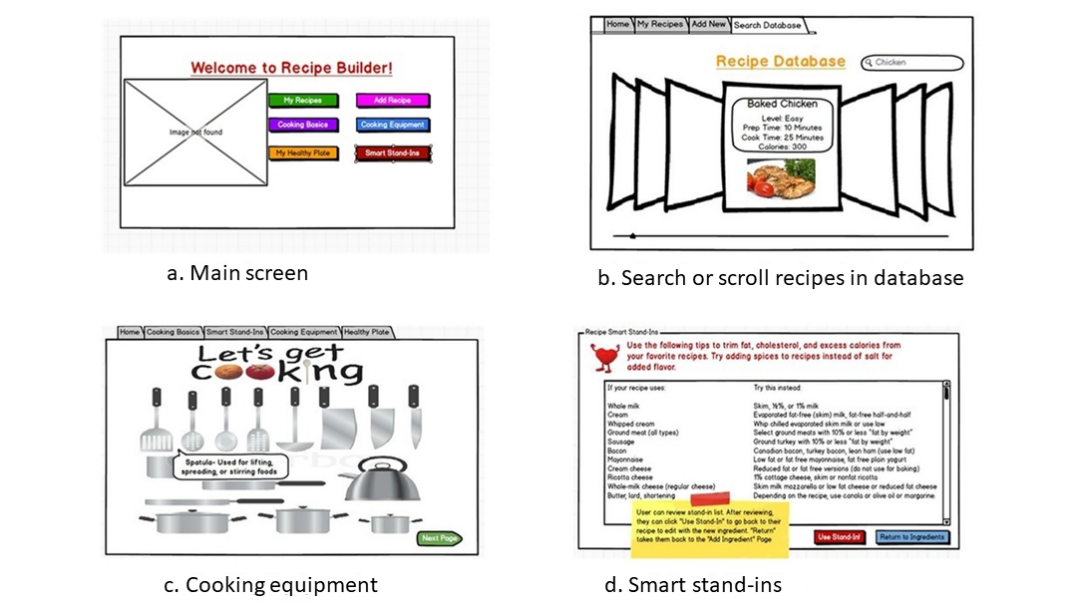
Culinary nutrition education-tech prototype screens.

Participants who reviewed the CNE prototypes evaluated 5 wireframe screen design prototypes (low- to midfidelity) [51] for the cooking app. The prototypes were presented to them on laptop, tablet, and mobile phones (see Figure 2 for example screens). The adolescents were also provided with paper printouts of the screen designs.

The general sessions included the following series of interactions and tasks:

- **Preinteraction With Screen Mock-ups**
  (1) Sessions began with interview questions regarding the adolescents’ use of technology, current cooking habits, and attitude toward using an app to assist with cooking.

- **During Interaction With Screen Mock-ups**
  (2) Think out loud review of the CNE-tech prototype.
  (3) Semantic differential scale [54] written assessment of the CNE prototypes.
  (4) Discussion of semantic differential scale responses with invitation to use markers, stickers, and other drawing supplies to identify issues and make changes to paper printouts of the prototype.

- **Postinteraction With Screen Mock-ups**
  (5) Detailed conceptual evaluation of the app.
  - Semistructured interview questions regarding the detailed design of the app for cooking.
  - Semistructured interview questions regarding the functions and general concept of using the app for cooking.
- (6) Semistructured interview questions regarding the context of use.

The usability session protocol further detailing each of the above activities is presented in Multimedia Appendix 1.

#### Interviews With Health Care Providers

During phase 2, we also conducted 10 semistructured, confidential telephone interviews (recorded audio tapes were destroyed after the study) with health care providers specialized in pediatric care and representing 4 states. The health care providers’ practices included care of overweight or obese adolescents. The pediatric endocrinologist that served on the larger project provided an initial convenience sample of health care providers for recruitment for the interviews. In addition, we expanded our interview pool through the use of snowball sampling [55].

The health care providers spoke from their personal experience with treating obese and overweight adolescents and their knowledge of health behavior programs and resources that aimed to assist patients facing this health issue at large. Physicians’ consent was received before arranging our interview sessions that lasted between 45 and 60 min. The health care providers received an electronic copy of the wireframed prototypes to reference during the call and the interviewer walked them through the app flow. The interview protocol focused on (1) their general reactions to the screen prototypes (the health care providers received the prototypes in advance), (2) challenges related to patient utilization of technology, (3) reactions to the prototypes, (4) their willingness to engage with technology, (5) ideas about the use and the integration of technology in summative patient reports in practice, and (6) opinions on the potential use of avatars and virtual agents. These interviews had limited coverage of the detailed design of each of the various app areas (eg, we did not go through preferred fonts and icons) as the health care providers took a more holistic assessment and recognized that they were not in a primary user role. Therefore, our results recognize specific statements directly or closely related to the CNE prototype and concepts, particularly related to safety and health issues; as well, the health care provider responses are most predominantly reflected in the context of use section of our results in recognition of their role in continuity of care.

### Data Analysis

After all usability walk-throughs were audio taped, transcribed, and reviewed for transcription errors, we conducted data analysis of deidentified transcripts using DEDOOSE, a qualitative data analysis application for data codification, classification, and treatment. Guiding principles proposed by Lee and Baskerville were applied to develop insights from the collected data [56].

We used constant comparison analysis to examine qualitative data [57,58]. First, 2 members of the research team independently coded interview transcripts and supplementary content. Our team deductively used the interview guide to formulate our high-level a priori coding schema (ie, predetermined coding). Researchers met regularly during this process to iteratively discuss emerging subcodes under each question and refine coding categories [59]. Intercoder disagreements regarding appropriate codes were resolved by consensus resolution, using an external qualitative expert to act as an auditor to make final determinations as needed. During the process, a few disagreements resulted from semantic issues of code name and precision, particularly whether or not to collapse detailed level coding into a higher code grouping. Consensus was ultimately reached among the 3 reviewers. We then carried out axial coding [59] to deductively collapse initial coding categories into specific functionalities and design requirements and contextual factors affecting use. Embedded in our interviewing and coding procedures, the validity and reliability of study data and interpretation were assessed following Lincoln and Guba’s criteria for evaluating interpretive research [60,61].

## Results

### Overview

Of the 15 adolescents who specifically participated in usability testing for the CNE-tech and responded to demographic, closed-ended questions preceding interaction with the prototypes, all 15 stated that they have access to a desk or laptop computer and a mobile phone, and 14 (14/15, 93%) have access to a tablet. Regarding the use of technology, respondents indicated that they used laptop and desktop computers primarily for taking notes, Web surfing, social networking, games, emails, and school work. Mobile phones were used for fitness apps, games, music, communication, social networking, and Web searching. Finally, the adolescents indicated that they used tablets mainly for social networking, games, and music.

Regarding cooking experiences, 11 (11/15, 73%) adolescents indicated cooking *often* or *sometimes* whereas only 4 (4/15, 27%) indicated *never cooking*. A total of 13 (13/15, 87%) adolescents indicated cooking with recipes. Of the adolescents, 6 (6/15, 40%) used recipes found by their family members, 3 (3/15, 20%) used recipes from books, 3 (3/15, 20%) found recipes on the Web, and 9 (9/15, 60%) stated that they created their own recipes. In response to open-ended questions about their cooking experiences, the adolescents brought up lack of knowledge about nutritional content, lack of time for cooking, challenges with remembering steps for meal preparation, and problems with using appliances. Following are the results of usability testing.

**Figure 3:**
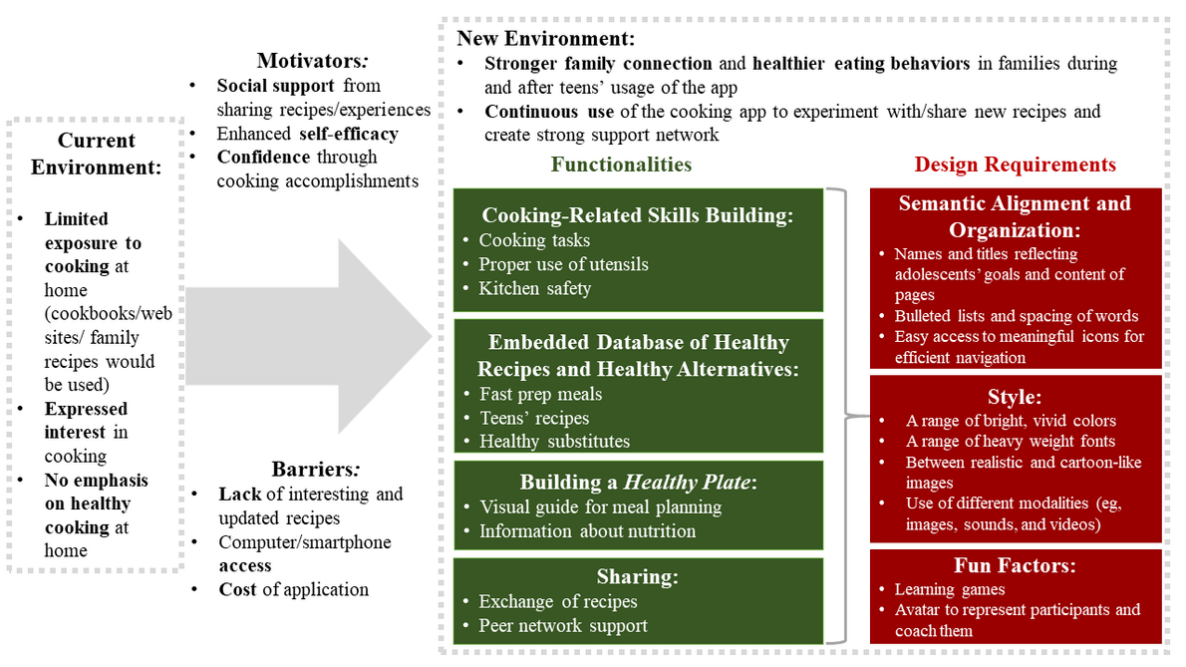
Summary of findings.

Figure 3 overviews our results from participant interaction with the prototypes (think out loud, semantic differential scale, and conceptual design interview questions to explore the details of functionalities and design) and subsequent discussion on situations of use to saturate our discussion on (1) CNE functionalities, (2) CNE design requirements, and (3) context of use.

**Figure 4:**
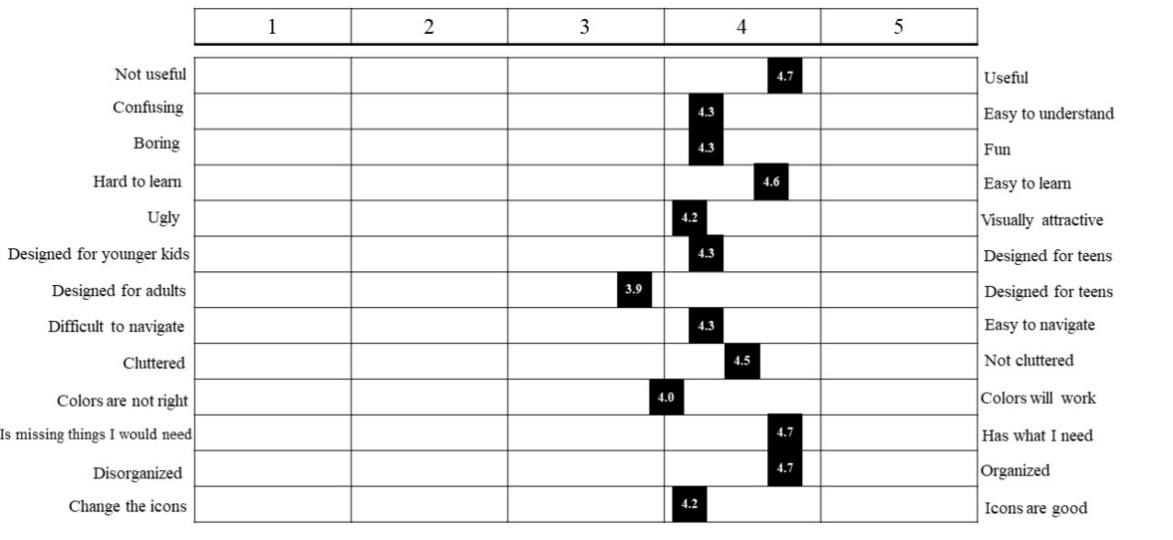
Semantic differential scale: the mean positions of adolescents.

Figure 4 summarizes the results from the semantic differential scale that the adolescents completed while interacting with prototypes. The figure displays the marked semantic differential scale for the adolescents showing the mean position on the scale. Overall, the aggregated results of this scale indicated that the high-level prototypes were positively assessed by the participants in terms of various constructs of the UTAUT model: the adolescents’ mean responses weighted toward useful, easy to understand, easy to learn, and aesthetic preferences, as demonstrated by the mean score on the semantic differential scale results from Figure 4. The results of this assessment also point to the relative attainment of the balance of *somewhere in between* childlike and adult design when it came to the look of the app. We extended the discussion of this balance into such areas as exploring appropriate icons and fonts to add to the evolving prototype, which we will discuss in the forthcoming design section.

### Key Functionalities

The adolescents indicated a series of functions that could be useful to facilitate their self-management as shown below.

#### Cooking-Related Skills Building

Many adolescents pointed out that they wanted to attain basic kitchen knowledge through the use of CNE-tech. They wanted to know *what knives to use, like what utensils you should use* and *what temperature to put the oven on*. Regarding specific cooking skills, they wanted to learn not just how to prepare the food but also creative ways to present the food. As 1 adolescent stated:

I would want to learn cool ways to prepare fruit like have you ever seen those design fruits where you put one fruit in the other fruit like it is some kind of a pattern. I think of creative ways to prepare food.

Moreover, the health care providers brought up the importance of kitchen safety:

People need to know how to defrost frozen foods, safely...They need to know how to use a knife safely. You need a little session on some of those in particular with the kids.

#### Embedded Database of Healthy Recipes and Healthy Alternatives

The adolescents stated that the CNE-tech should contain a stocked database of predetermined healthy recipes. They also indicated that the database should include recipes for all meal times and snacks and that every recipe should include estimated preparation times (ideally short) because of their tight schedules by stating “the recipes need to not take a lot of time, thirty minutes or less, if possible. You know, it needs to involve low-cost ingredients.” They further indicated that recipe metainformation should include complexity level and nutritional information. Furthermore, the adolescents indicated that they wanted pictures to accompany step-by-step instructions.

In addition to prepopulated recipes, adolescents also wanted to be able to add their own recipes to the database. In making these additions or changes, adolescents wanted healthy guidance, namely, that the app would suggest substitutions for unhealthy ingredients when building or reviewing a recipe. As one of them stated:

Because I do alter up a lot of things when I’m cooking and substitute it for different things. Also, it’ll be helpful to have the healthier substitutions thing going so that way I can kind of be like, “Yay healthy.”

This preference for guidance extended to some adolescents suggesting alerts in case of an *unhealthy choice*. Furthermore, the participants suggested functionality that would convert recipes they found outside of the app to healthy ones leveraging these substitutes. As stated by one adolescent:

I would absolutely love it if there was an app, that you could plug in your recipes, you know, even just a normal recipe that you haven’t modified to make healthy, you just want to know.

One participant suggested that the CNE-tech could have links to specific external websites that would allow them to pick and convert recipes into healthier alternatives.

Regardless of the original source of the recipe, many adolescents indicated the desire to add personal notes and pictures of meals they prepared to the recipes in their database.

#### Building a Healthy Plate and Learning About Nutrition

Given that the adolescents were already in a program making their way toward behavior change, it was not surprising that they understood the concept of a healthy meal and attention to daily food consumption. To help themselves aim for a healthy meal in selecting recipes, the adolescents described a plate metaphor, a proportional puzzle, that the app would consider aggregated food choices to help them balance their meals. A proportional plate function was referenced as a visual guide for meal planning that would indicate the missing nutritional elements and provide suggestions to complete building a healthy plate.

The adolescents also indicated that the CNE-tech could provide a means to learn more about nutrition:

[We can learn] the nutrition about the little star next to things that basically says it’s a 2000 calorie diet, when you know most people were on a 1500 calorie diet.

#### Sharing

The adolescents mentioned that they wanted the CNE to have a private peer network that could enable sharing and posting pictures or information about recipes and their experiences with family and friends and camp counselors. One of the participants said:

I don’t normally share recipes on Facebook, but with this site, I probably might because they are going to be healthy and then I can show my friends “Oh, look I am eating healthy you should to.”

The adolescents indicated that this peer sharing of cooking and eating accomplishments would motivate them to eat and cook healthier.

### Key Design Requirements

The adolescents expressed a series of preferences in technology design particularly related to alignment between names and content, page layouts, colors, fonts, image styles, use of modalities, games, and avatars.

#### Semantic Alignment and Organization

The adolescents were sensitive to semantic alignment of the app name and headers. They preferred that the name of the app reflect the goal of the app (ie, *Health Life, Health Track*, and *Nutrition Buddy*) and titles of individual pages match the content of such pages.

Furthermore, the participants indicated that the content within pages should consider the use of bulleted lists, white space and other organizing structures to present information clearly. A tabular structure for organizing recipes was mentioned:

A tab for...recipes because there would be a lot of them right, then you could have my friend’s recipes as another tab.

As for the overall layout of the app, the adolescents wanted easy access to organized and meaningful icons to facilitate efficient navigation. As one of them stated:

When you open it, it can have your different choices at the bottom of a screen, and one of them could be that icon for restaurant, and then another one for tips or something, and then another one for the game, and then another one for whatever else we want.

#### Style

**Figure 5:**
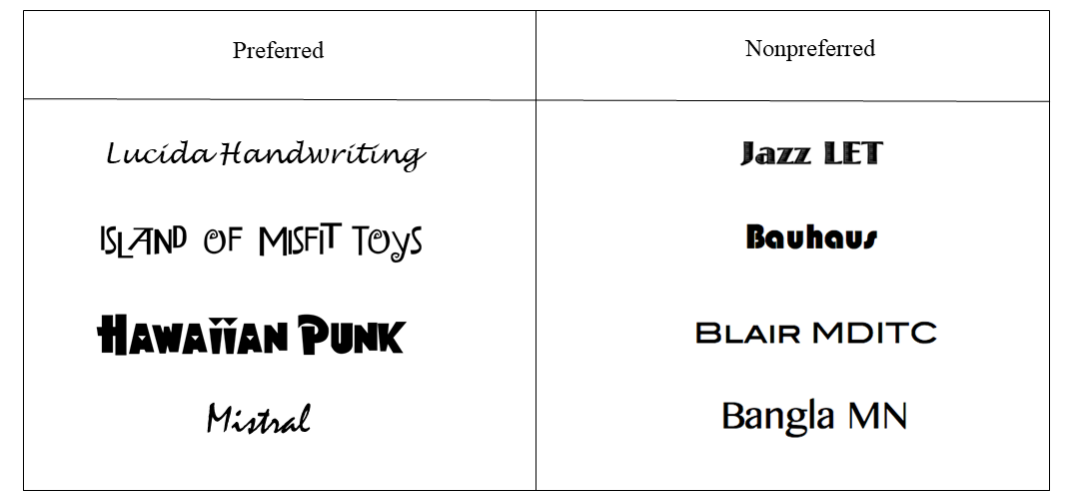
Examples of fonts.

The adolescents expressed enthusiasm about having a range of bright, vivid colors throughout the app. They also indicated that they wanted to choose among different fonts and were particularly interested in font types, such as Comic Sans, Custom Bubble, Hawaiian Punk, Disco, Island of Misfit Toys, Lucida Handwriting, Montgomery, and Princetown LET, Star Guide; research studies indicate that these fonts (which fall into the script or funny category established in past studies) convey a happy and creative emotional message [62]. Figure 5 shows examples of contrasting font preferences. The participants also noted that the fonts chosen should be consistent throughout the app to unify it.

**Figure 6:**
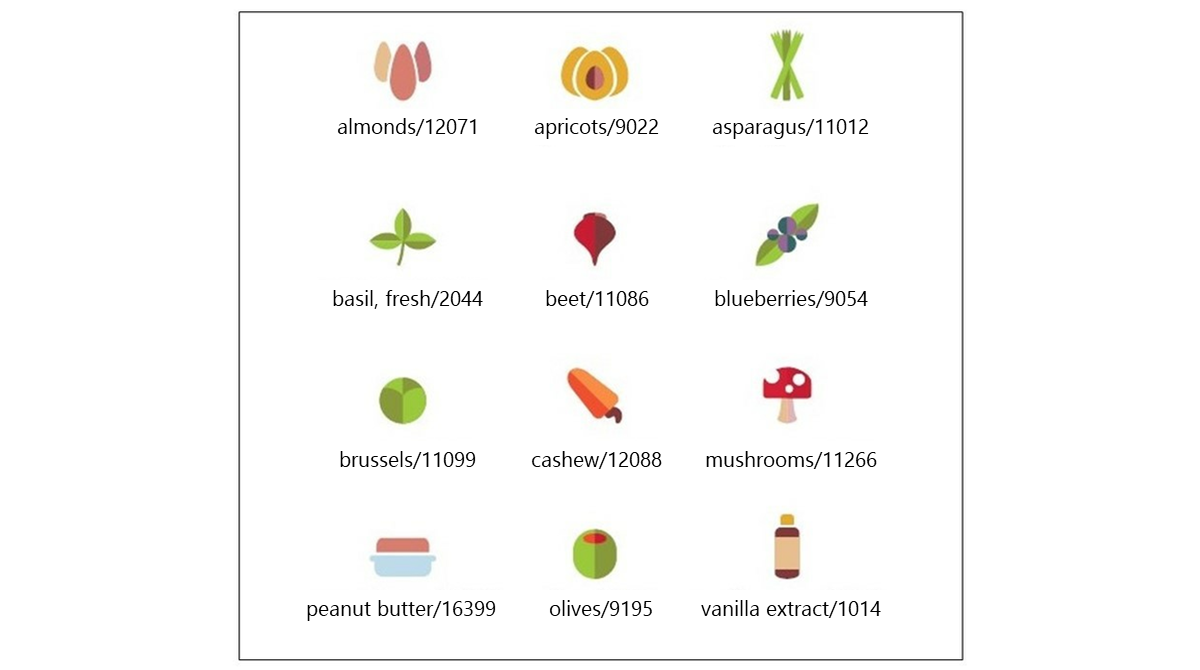
Example of the final design of the food images.

The adolescent responses to image styles indicated that a careful balance between realism and playful was needed—semireal cartoon. Figure 6 contains examples of the final design for the images to strike a balance between adult and childlike based on the adolescents’ responses from a semantic differential scale described previously and their responses to various style image options. To present these images, some participants indicated a preference of borders around the images and further specified a preference for circular (rounded corner) rather than rectangular shaped borders within the CNE-tech.

The participants also expressed their interest in the use of different modalities, such as images, sounds, and videos, within the app. They indicated that these modalities could complement and enliven various app areas, for example, interactive video clips could accompany challenging recipes. As for sounds, the adolescents suggested sound effects that resemble real sounds related to cooking, as 1 adolescent mentioned:

When something is in the oven, it will be, like sizzle too, but louder ones. And when you’re washing your hands, shhh, like that sound. And when you’re cutting (pounding on the table) sound.

#### Fun Factors: Gaming and Avatars

The adolescents also revealed their interest in the gaming component of the app. Learning games (ie, identifying unhealthy food options) were brought by the participants, meaning that they wanted the gaming component to tie to the learning objective of the app, cooking literacy. One of the participants stated:

I think that it defeats the purpose of a website that helps kids, the games would be just something they would play for fun, it wouldn’t be really helping their health or unless they were like inspired by the game, but I don’t see that happening.

Finally, the adolescents indicated that avatars could serve multiple purposes in the app (eg, animated representation of the participants or a coach demonstrating cooking tasks). The participants indicated that avatars could bring some fun factors into the app; as one of them said:

You could favorite one of your recipes and then when you got close to one, it [Avatar] would jump [up and] down silently.

### Evolved Prototypes

**Figure 7:**
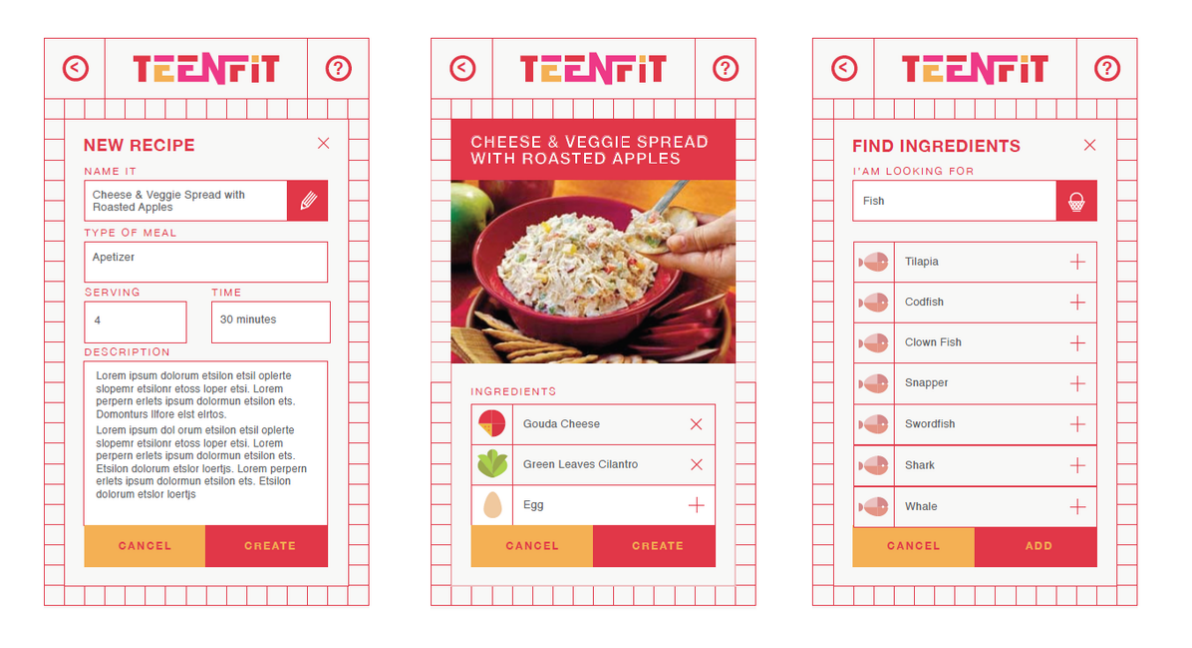
Modified screen designs.

Figure 7 provides examples of the evolved screen design for a mobile phone platform based on the design sessions with the adolescents.

### Context of Use

The CNE-tech was of high interest to all parties involved in this study. The health care providers expressed particular enthusiasm, as one of them stated,

It never really crossed my mind because, of course, my boys like to cook and they wanted to learn from us, and then we are really cooking so, that actually never really crossed my mind that these kids when they leave home don’t know how to cook, so they will be more likely [to eat] fast foods and stuff like that.

Furthermore, the parents engaged in phase 1 of the study thought that the CNE-tech that could help their adolescent was beneficial and fun:

Something to help your child with meal plans?

Interviewer

Yeah, I think they would definitely use that because they are very computer and internet savvy. They would consider that fun I think. They would have control over choices and decisions.

Parent

The adolescents were excited about cooking in general, as some of them already cooked using recipes from family, books, websites, and even food packages. They also revealed that sometimes they created their own recipes for food items, while acknowledging that these experiments were not always successful, as 1 participant said:

I’ve tried, but they’re kind of a train wreck. They don’t taste very good either, but I try though.

When the adolescents compared this app with the existing cookbooks and other websites or apps, they pointed out some differences from what they used, such as having recipes in one place, calorie counting, and nutritional information that is simple and comprehensive. As 1 participant indicated:

So this is a lot like **Epicurious** but it is a little more easy to understand and it gives you substitutes if you do not want to use what it says.

According to the adolescents and their parents, lifestyle management that involves tracking food intake and cooking ability would improve as a result of using the app. The adolescents indicated they would cook more with the CNE-tech. They also recognized that using the CNE-tech to enact healthy cooking and eating would be part of their self-management. One of the participants said:

I think it would work because then you can kind of see exactly what you’re putting into your body. If your doctor says, “Oh, you need more calcium” or something, you would know that your food has calcium in it.

Some of the parents, for example, were particularly interested in the healthy calorie intake per meal, as 1 of them brought up:

Yeah, what am I looking at here? How many grams of fat would be in this? How many grams of sugar? How many calories? You know, what would be a decent serving size of this to stay within a decent amount of calories?

Furthermore, the adolescents were particularly enthusiastic about the option of sharing their progress and recipes with their social network (specific mention included parents, siblings, grandmother, friends, camp counselors, and doctors). One of the adolescents said:

Well I’d like my friends and my family to go [into the app] and they can go in separately and alone, but I could go on with my mom and it might be that, “Hey. Do you want any of recipes” and then she can help me look at different stuff.

Such aspects of social sharing of recipes were also brought up by the health care providers in the interviews:

I think that regarding the recipe, like if they create a recipe, or choose a family recipe or whatever that they adding to it, there will be a way that they could share that with their social group and all the social age kind of stuff.

The participants were optimistic to use the CNE-tech every day around dinner time. One of the adolescents said:

I’d have time to use it, probably, I’d try to use it every night just to see if I could make something or create something.

Furthermore, some adolescents stated that they would generally use healthy behavior technology multiple times a day during the first month and keep using it afterward. The parents were supportive of such an idea and expressed willingness to work with their children on recipes. One of the parents stated:

Her having the app so that I can say, “Hey, find something that’s approved on the app, yeah, well go have it let’s do it.” So, she has resources to find things that she likes, or recipes or ways to cook her favorite foods that she can give to me.

The adolescents indicated that their usage, however, might decline because of the lack of interesting and updated recipes or lack of information on healthy alternatives and substitutions. As one of them stated:

But one of the times where I might be less interested in the website would be if the recipes were getting less interesting or if it is starting to become stuff that I didn’t like.

In addition, potential barriers, such as access to technology or cost of technology could affect usage.

## Discussion

### Behavioral Change Theory Alignment

Eating home-cooked meals along with the recommended amounts of fruits and vegetables is associated with an increase in culinary skills and healthy cooking behaviors that could combat obesity and overweight [5]. Our results demonstrate the functional needs and design preferences from stakeholders (eg, adolescents, parents, and health care providers) for a CNE-tech that focuses on sustainable nutrition planning while capturing the fun, rewarding side of cooking. We then identified contextual factors, such as strong support system, social network, and motivators to increase self-esteem and self-efficacy, that could affect the participants’ use of CNE-tech and sustain usage thereof over time.

Understanding requirements for the design and functionalities of the CNE-tech provides us with certain insights into the mental models of our target users, so we have a better chance of creating a technology-enabled solution that would appeal to our audience. In addition to practical implications, our results demonstrated some alignment with the existing behavioral change theories, meaning that identified requirements exhibited high potentiality to induce changes in the participants’ health behaviors.

Our functional and design guidelines echo various aspects of behavioral change theories. Reflection of various constructs from behavioral change theories in mobile app interventions is not a novel idea, as it was demonstrated in the recent review on commercially available cooking apps in the Apple store [38]. Highly ranked diet tracking apps emphasize behavioral strategies, such as perceived social norms, self-monitoring, and realistic goal setting [63]. In our study, functionalities and design requirements are also tied to similar constructs on which behavioral strategies might be grounded.

The adolescents’ preferences toward various elements of design requirements and functionalities appear to align with constructs of the Social Cognitive Theory (SCT), one of the main behavior change theories focusing on the ongoing change in a person’s behavior [64], and the Fogg Behavior Model (FBM), which explores the effect of persuasive design on people’s behaviors [65].

In the SCT, the concept of reciprocal causation implies that there exists an ongoing, impactful interaction among environment, person, and behavior [64]. Our findings have shown that the adolescents would be eager to become more responsible for family meal preparation as they build up their food literacy using our CNE-tech. Exposing their families to dietary changes, the adolescents may alter their environment by effecting their relationships with their families along with healthy habits of their families. As the parents become more involved in dietary changes of their children (eg, buying ingredients, assisting the adolescents with certain cooking tasks, or participating in social and support networks), they will spend more time with the adolescents, build stronger family connections over time, and adopt healthy eating behaviors.

Before they induce any change in their environment and their own lifestyle, the adolescents need to have necessary skills to do so. Their behavior capability, referring to skills mastery in the SCT [64], would be enriched by cooking-related skill building (ie, learning about basic utensils or creating their own recipes). Design of pages and embedded database of healthy recipes in the CNE-tech prototype align with the concepts of ability from the FBM [65]. According to Fogg, ability in the persuasive technology implies the power of simplicity that makes behavior easier to do. This concept was reflected in highly organized, step-by-step recipes and easy-to-use embedded database of healthy recipes desired by the adolescents.

Trigger, another component of the FBM, is a vital element of persuasive technology that functions to activate a person’s behavior by, for example, inspiring hope or highlighting fear [65]. This construct was reflected in the adolescents’ requirements for the naming of app and page titles that should evoke the adolescents’ vision of their future self.

Design requirements also show the desire for the extensive use of multimedia, such as pictures, audios, and videos, and such requirements align with the concept of observational learning from the SCT [64], meaning that adolescents are eager to learn by watching and listening to others in the lessons. Different elements of the interface, either bright colors in images or realistic sounds in videos, should create some kind of a happy, fun, and energetic feeling, yet they should not be too childish for adolescents. All combined, these elements should contribute to forming and maintaining adolescents’ weight goals and expectations, another important component of the SCT [64].

One of the major constructs in the SCT is self-efficacy, a cognitively based source of motivation that encourages people to persist in their efforts [66]. After accomplishing a given level of performance, adolescents may no longer be satisfied with their progress level and take further steps toward higher attainments. This aspect of building self-efficacy was indicated by their general interest to use the CNE-tech and the gamification part, which would allow the adolescents to set incremental goals and build their self-efficacy in a fun, yet challenging way. Gamification along with using an avatar can also induce internal reinforcement [64] of adolescents’ behavior as they reward themselves by building up their cooking confidence and undertaking more challenging recipes.

External reinforcement [64] of adolescents’ behaviors stems from their participation in social and support networks. As adolescents share their experiences with and receive feedback from their family and friends, they might be exposed to social persuasion, which can contribute to the successful outcomes achieved through corrective performance, in the form of verbal encouragement [66]. In the FBM, Fogg discusses the idea of motivation in the form of social acceptance [65], which seems to align with adolescents’ use of social and support networks where they share their experiences and may feel more socially accepted by their peers and families as they continue their journey toward building healthy habits.

### Practical Implications

Our results bring to light aspects of functional and design misalignment that some existing cooking and weight management technologies have with overweight adolescents’ needs and preferences for maintaining healthy eating. For example, some existing apps record users’ data, but focus primarily on the number of consumed calories rather than nutritional value of foods. Our data indicate that adolescents need and want to know more about food and cooking than calories. A cooking app with extended information can introduce the adolescents to nutrient-dense foods. Adolescents can use CNE-tech to not only cook at home and at the home of someone in their network but also guide their food choices in other settings. Benefits can be gained from away-from-home meals, such as packing lunches for school or selecting healthier food choices in restaurants or school cafeterias when options are available. Moreover, some programs on the market focus on selling branded, prepared meals. Such programs could provide customers with a healthy balance, yet failed to teach food literacy, which was clearly supported by the study participants. It seems that many available cooking apps expect users to have at least some experience in cooking, limiting their applicability to adolescents, who seem to be novices in many cases. Therefore, adolescents want the app to start from the basics (eg, cutting, frying, using utensils, and understanding oven temperatures) and help them improve their cooking skills over time. Current cooking apps, however, seem to overall lack such functions and the preferred design, creating the need for tools that embody the results of this study.

### Limitations

Our study is subject to various limitations affecting generalizability. Identified functionalities and design preferences could be more relevant to the segment of adolescent population that participated in our study, rather than to the overall population of overweight adolescents. For both phases of this study, our focus was on looking at healthy behavior technologies to extend punctuated or short-lived programs. This means that adolescents who participated in the study represented a population of adolescents enrolled in healthy behavior programs, meaning that they and their parents were aware of the weight issue and understood the need for behavioral modification. These adolescents were actively seeking change, and readers should recognize that our study might again not be generalizable to other overweight or obese adolescents in the country, who may not yet be receptive to or actively seeking change. Furthermore, the study is based on the assumption that adolescents are already using technologies and will be doing so in the future. Such assumption might not hold in some contexts, as some families might be more restrictive about their children using technologies or the capacity of the technologies the adolescents have access to might be incompatible with the CNE-tech for some reason.

Another assumption that might not hold is the access to healthy food options. CNE-tech might be challenging to use in communities that have limited access to healthy and nutritious food because of financial and other constraints. There, however, exist public health programs, such as the Healthy Food Financing Initiative and Supplemental Nutrition Assistance Program [67], that aim to provide the underserved communities with access to healthy and affordable food options.

It is also of note that to extend further reach of these technologies and programs to those in lower socioeconomic status, iterations of an app informed by the larger study are under development. The effort is specifically targeted to extend the reach of the evidence-based healthy programs to adolescents who are not able to attend these programs because of various limitations and socioeconomic constraints. The concept will be tested in late 2019 and will couple functionalities of the technology with virtual coaching and guidance from professionals involved in the on-site evidence-based healthy behavior programs.

Future research may aim to address the above-mentioned limitations and the role of national public health initiatives to support generalizability of our results.

### Conclusions

As adolescent overweight and obesity collectively constitute one of the major health issues in our population [1], we need to understand the importance of behavioral health interventions that could cause a sustainable change in self-care. The overall decline of culinary skills and home cooking in the US population stands in the way of combating obesity and overweight and associated health conditions. Our study leveraged a multistakeholder (eg, adolescent, parent, and provider) user-centered approach to tailor CNE-tech requirements and design to adolescents’ preferences and needs and also identified contextual factors affecting the potential use. Overall, our study indicates that adolescents, health care providers, and parents see that CNE-tech solutions that address the features they specified have the potential to facilitate the self-management of overweight and obesity. The culinary skills acquired may be additive or exponential as upon achieving a critical level of food literacy, an overweight adolescent can not only prepare food for themselves but also share healthy meals, teach others to cook, and actively advocate for their own healthy nutrition needs. Furthermore, the acquisition of food literacy and cooking confidence might have some carryover effects to other forms of self-management and self-esteem.

## Appendix

Multimedia Appendix 1 User centered design session protocol.

## Abbreviations

CNE: culinary nutrition education
FBM: Fogg Behavior Model
ICT: information and communication technology
SCT: Social Cognitive Theory
UCD: user-centered design
UTAUT: Unified Theory of Acceptance and Use of Technology

## References

[1] Carroll MD, Navaneelan T, Bryan S, Ogden CL (2015). Centers for Disease Control and Prevention.

[2] Al-Goblan AS, Al-Alfi MA, Khan MZ (2014). Mechanism linking diabetes mellitus and obesity. Diabetes Metab Syndr Obes.

[3] You J, Choo J (2016). Adolescent overweight and obesity: links to socioeconomic status and fruit and vegetable intakes. Int J Environ Res Public Health.

[4] Hruby A, Hu FB (2015). The epidemiology of obesity: a big picture. Pharmacoeconomics.

[5] Güngör NK (2014). Overweight and obesity in children and adolescents. J Clin Res Pediatr Endocrinol.

[6] Sahoo K, Sahoo B, Choudhury AK, Sofi NY, Kumar R, Bhadoria AS (2015). Childhood obesity: causes and consequences. J Family Med Prim Care.

[7] Anderson PM, Butcher KE (2006). Childhood obesity: trends and potential causes. Future Child.

[8] Utter J, Larson N, Laska MN, Winkler M, Neumark-Sztainer D (2018). Self-perceived cooking skills in emerging adulthood predict better dietary behaviors and intake 10 years later: a longitudinal study. J Nutr Educ Behav.

[9] Wolfson JA, Frattaroli S, Bleich SN, Smith KC, Teret SP (2017). Perspectives on learning to cook and public support for cooking education policies in the United States: a mixed methods study. Appetite.

[10] Okrent AM, Kumcu A (2016). Economic Research Service.

[11] Siddiqua AA, Alem MS (2018). Impact of situational factors in students' preference of fast food - an empirical study. Int J Res Com Manag.

[12] Contini C, Boncinelli F, Gerini F, Scozzafava G, Casini L (2018). Investigating the role of personal and context-related factors in convenience foods consumption. Appetite.

[13] Saghaian S, Mohammadi H (2018). Factors affecting frequency of fast food consumption. J Food Dist Res.

[14] Janssen HG, Davies IG, Richardson LD, Stevenson L (2018). Determinants of takeaway and fast food consumption: a narrative review. Nutr Res Rev.

[15] (2015). Region of Waterloo.

[16] Raber M, Chandra J, Upadhyaya M, Schick V, Strong LL, Durand C, Sharma S (2016). An evidence-based conceptual framework of healthy cooking. Prev Med Rep.

[17] Perera T, Frei S, Frei B, Wong SS, Gerd B (2015). Improving nutrition education in US elementary schools: challenges and opportunities. J Educ Pract.

[18] Snyder SA, Dougall AL (2018). Assessment of nutrition topics for education in college-aged adults. J Food Nutr Res.

[19] Kris-Etherton PM, Akabas SR, Bales CW, Bistrian B, Braun L, Edwards MS, Laur C, Lenders CM, Levy MD, Palmer CA, Pratt CA, Ray S, Rock CL, Saltzman E, Seidner DL, van Horn L (2014). The need to advance nutrition education in the training of health care professionals and recommended research to evaluate implementation and effectiveness. Am J Clin Nutr.

[20] Guthrie JF (2017). Integrating behavioral economics into nutrition education research and practice. J Nutr Educ Behav.

[21] McMullen J, Ickes M (2017). The influence of a campus-based culinary, nutrition education program, 'College CHEF,' on college students' self-efficacy with cooking skills and nutrition behaviors. Build Healthy Acad Communities J.

[22] Kerrison DA, Condrasky MD, Sharp JL (2017). Culinary nutrition education for undergraduate nutrition dietetics students. Brit Food J.

[23] Abraham S, Noriega B, Shin JY (2018). College students eating habits and knowledge of nutritional requirements. J Nutr Hum Health.

[24] Thomas HM, Irwin JD (2011). Cook it up! A community-based cooking program for at-risk youth: overview of a food literacy intervention. BMC Res Notes.

[25] Wrieden WL, Anderson AS, Longbottom PJ, Valentine K, Stead M, Caraher M, Lang T, Gray B, Dowler E (2007). The impact of a community-based food skills intervention on cooking confidence, food preparation methods and dietary choices - an exploratory trial. Public Health Nutr.

[26] Bernardo GL, Jomori MM, Fernandes AC, Colussi CF, Condrasky MD, Proença PR (2018). Positive impact of a cooking skills intervention among Brazilian university students: six months follow-up of a randomized controlled trial. Appetite.

[27] Utter J, Denny S, Lucassen M, Dyson B (2016). Adolescent cooking abilities and behaviors: associations with nutrition and emotional well-being. J Nutr Educ Behav.

[28] McMullen J, Ickes M, Noland M, Erwin H, Helme D (2016). Development of 'College CHEF,' a campus-based culinary nutrition program. Am J Health Educ.

[29] do Amaral EM, de Carvalho SV, dos Santos CC, Toral N (2017). Nutritional interventions for adolescents using information and communication technologies (ICTs): a systematic review. PLoS One.

[30] Free C, Phillips G, Galli L, Watson L, Felix L, Edwards P, Patel V, Haines A (2013). The effectiveness of mobile-health technology-based health behaviour change or disease management interventions for health care consumers: a systematic review. PLoS Med.

[31] (2015). Research 2 Guidance.

[32] Lee SY (2014). Examining the factors that influence early adopters’ smartphone adoption: the case of college students. Telemat Inform.

[33] Wartella EA, Rideout V, Montague H, Beaudoin-Ryan L, Lauricella AR (2016). Teens, health and technology: a national survey. Media Commun.

[34] Anderson M, Jiang J (2018). Pew Research Center.

[35] Majeed-Ariss R, Baildam E, Campbell M, Chieng A, Fallon D, Hall A, McDonagh JE, Stones SR, Thomson W, Swallow V (2015). Apps and adolescents: a systematic review of adolescents' use of mobile phone and tablet apps that support personal management of their chronic or long-term physical conditions. J Med Internet Res.

[36] Stein K (2015). Remote nutrition counseling: considerations in a new channel for client communication. J Acad Nutr Diet.

[37] Chen J, Lieffers J, Bauman A, Hanning R, Allman-Farinelli M (2017). The use of smartphone health apps and other mobile health (mHealth) technologies in dietetic practice: a three country study. J Hum Nutr Diet.

[38] Azar KM, Lesser LI, Laing BY, Stephens J, Aurora MS, Burke LE, Palaniappan LP (2013). Mobile applications for weight management: theory-based content analysis. Am J Prev Med.

[39] Wei J, Cheok AD, Martinez XR, Tache R, Zhu Q (2011). Foodie: Play With Your Food Extend Social Cooking Game With Novel Edible Interface. Proceedings of the International Games Innovation Conference.

[40] Thompson D, Bhatt R, Vazquez I, Cullen KW, Baranowski J, Baranowski T, Liu Y (2015). Creating action plans in a serious video game increases and maintains child fruit-vegetable intake: a randomized controlled trial. Int J Behav Nutr Phys Act.

[41] Hingle M, Patrick H (2016). There are thousands of apps for that: navigating mobile technology for nutrition education and behavior. J Nutr Educ Behav.

[42] Pagliari C (2007). Design and evaluation in eHealth: challenges and implications for an interdisciplinary field. J Med Internet Res.

[43] LeRouge C, Wickramasinghe N (2013). A review of user-centered design for diabetes-related consumer health informatics technologies. J Diabetes Sci Technol.

[44] Michie S, Yardley L, West R, Patrick K, Greaves F (2017). Developing and evaluating digital interventions to promote behavior change in health and health care: recommendations resulting from an international workshop. J Med Internet Res.

[45] Merriam-Webster.

[46] Lyon AR, Koerner K (2016). User-centered design for psychosocial intervention development and implementation. Clin Psychol (New York).

[47] Venkatesh V, Morris MG, Davis GB, Davis FD (2003). User acceptance of information technology: toward a unified view. MIS Q.

[48] Tong A, Sainsbury P, Craig J (2007). Consolidated criteria for reporting qualitative research (COREQ): a 32-item checklist for interviews and focus groups. Int J Qual Health Care.

[49] Knoblock-Hahn AL, Wray R, LeRouge CM (2016). Perceptions of adolescents with overweight and obesity for the development of user-centered design self-management tools within the context of the chronic care model: a qualitative study. J Acad Nutr Diet.

[50] Knoblock-Hahn AL, LeRouge CM (2014). A qualitative, exploratory study of predominantly female parental perceptions of consumer health technology use by their overweight and/or obese female adolescent participating in a fee-based 4-week weight-management intervention. J Acad Nutr Diet.

[51] Engelberg D, Seffah A, Hammond J, Gross T, Wesson J (2002). A framework for rapid mid-fidelity prototyping of web sites. Usability: Gaining a Competitive Edge.

[52] Camp Jump Start: Weight Loss Camp for Teens - More than a Fat Camp.

[53] Huelsing J, Kanafani N, Mao J, White NH (2010). Camp jump start: effects of a residential summer weight-loss camp for older children and adolescents. Pediatrics.

[54] Frey B (2018). The Sage Encyclopedia of Educational Research, Measurement, and Evaluation. Volume 1-4.

[55] Berg S (2002). Snowball sampling. Encyclopedia of Environmetrics.

[56] Lee AS, Baskerville RL (2003). Generalizing generalizability in information systems research. Inform Syst Res.

[57] Glaser BG (1965). The constant comparative method of qualitative analysis. Soc Probl.

[58] Hsieh HF, Shannon SE (2005). Three approaches to qualitative content analysis. Qual Health Res.

[59] Saldana J (2013). The Coding Manual for Qualitative Researchers. Second Edition.

[60] Guba EG (1981). Criteria for assessing the trustworthiness of naturalistic inquiries. Educ Technol Res Dev.

[61] Guba EG, Lincoln YS (1985). Naturalistic Inquiry. First Edition. Volume 75.

[62] Shaikh D, Chaparro B (2016). Digital font and reading. Series on Language Processing, Pattern Recognition, and Intelligent Systems.

[63] Doshi A, Patrick K, Sallis JF, Calfas K (2003). Evaluation of physical activity web sites for use of behavior change theories. Ann Behav Med.

[64] Bandura A (1986). Social Foundations Of Thought And Action: A Social Cognitive Theory.

[65] Fogg BJ (2009). A Behavior Model for Persuasive Design. Proceedings of the 4th International Conference on Persuasive Technology.

[66] Bandura A (1977). Self-efficacy: toward a unifying theory of behavioral change. Psychol Rev.

[67] Let's Move!.

