# Supplementary material for: Design Guidelines for a Technology-Enabled Nutrition Education Program to Support Overweight and Obese Adolescents: Qualitative User-Centered Design Study
Source: J Med Internet Res. 2019 Jul 29;21(7):e14430. doi: 10.2196/14430 (PMC6690165; doi:10.2196/14430)
Supplement: Multimedia Appendix 1 [file jmir_v21i7e14430_app1.pdf]

Multimedia Appendix 1: User Centered Design Session Protocol

This is a Multimedia Appendix to a full manuscript in the J Med Internet Res. For full copyright and citation information see http://dx.doi.org/10.2196/14430.

Pre-task questions – Current Practices

You are going to review the recipe builder section of this application idea. Let's first learn a bit about what you do now.

1. Do you generally cook?
2. Do you generally cook with recipes?
3. Where do you get your recipes?
4. Do you create your own recipes?
5. What is difficult for you to currently cook your own food?
6. Do you have a computer? iPad? IPhone? What types of things do you use it for?
7. Generally, what do you think about the idea of using a computer to help you use recipes to cook?

Task - Think out loud interaction with prototypes

I am now going to let you take a look at the rough screen designs (mock ups, initial thoughts) based on the ideas provided by teens last summer. We would like to hear your thoughts about these to know if this is on track. I am going to give you some time to take a look and provide you with a question sheet that will help guide our discussion.

Please speak out loud and let me know your impression and thoughts as you go through the application. Feel free to indicate anything you might want to change or add as you go along. You can state the changes verbally. In addition, you can mark/draw or add stickers to the paper printout.

Semantic Differential Scale - While Interacting with Prototypes

	1	2	3	4	5	
Not useful						Useful
Confusing						Easy to understand
Boring						Fun
Hard to learn						Easy to learn
Ugly						Visually attractive
Designed for younger kids						Designed for teens
Designed for adults						Designed for teens
Difficult to navigate						Easy to navigate
Cluttered						Not cluttered
Colors are not right						Colors will work
Is missing things I would need						Has what I need
Disorganized						Organized
Change the icons						Icons are good

The participants were asked to put comments for items scored at a 3 or below. Some iteration of the following questions:

- How would you change this?
- What don't you like?
- What would help to move the score up to a five?
- What would you add?
- What would you remove?

Encourage the teen to show you what they would change by modifying the print outs.

Post-task questions *Concept Evaluation:*

General Fit:

1. What do you think about using this specific application to help you cook healthy?
2. What parts of the applications are similar to what you do currently?
3. What is different?
4. How can this work with what you are trying to do according to your doctor's advice and what you are trying to learn from your healthy living program (i.e. Camp Jump Start)?

Design:

1. What would make this application easier to use?
2. What were the things you liked most about the application screens you viewed?
3. What needs to change on this application to make it better?

I am going to let you review some stickers and pictures that could be potential icons and pictures in this application (show appropriate picture pack and/or stickers). Do not worry so much about the actual picture, but of the style of picture and tell me which of these types of pictures do you think will work in this application by circling those and put an X through things you just do NOT like. Let me know if you like any of these better than the types of images you see in the application now. We will cut those out and past them on the application sheet.

4. Here are some additional font options. Do you like any of these options better than what is currently in the application? (Show examples of the fonts and circle the ones they like.)
5. Is there anything missing from the applications that could be added to help you manage a healthy lifestyle? Yes/No If yes, what is?
6. Last summer some teens indicate that they liked avatars. Do you think this application is a good place to add an avatar?
7. Some applications now are in the form of a game. Would you like to turn this application into a game of some sort? What would the game be like? How could you score or get rewarded in the game?
8. The current screen designs are for a PC. What part of this application would you want on a smart phone or iPad? Take a look at this tablet (or phone). Keep in mind screen space is limited on a smart phone and iPad.
9. Would you really use the part you want on a smart phone or iPad as you go around?

Post-task questions *Context of Use:*

10. Are there any problems that may cause you not to use the applications you reviewed?
11. When do you think these applications would be most helpful to you?
12. How would you want them to use the application with you?
13. Would you want anyone to use this application with you? Is there anyone you would like to share your information from this application with?
14. How often would you want to use these applications within the first month of getting them? What about after that?
15. What would motivate you use this application for three consecutive months?
16. Are there any other types of applications that you think would help you with healthy living?
